# Supplementary figures and images for: Mycobacterium ulcerans challenge strain selection for a Buruli ulcer controlled human infection model
Source: PLoS Negl Trop Dis. 2024 May 3;18(5):e0011979. doi: 10.1371/journal.pntd.0011979 (PMC11095734; doi:10.1371/journal.pntd.0011979)

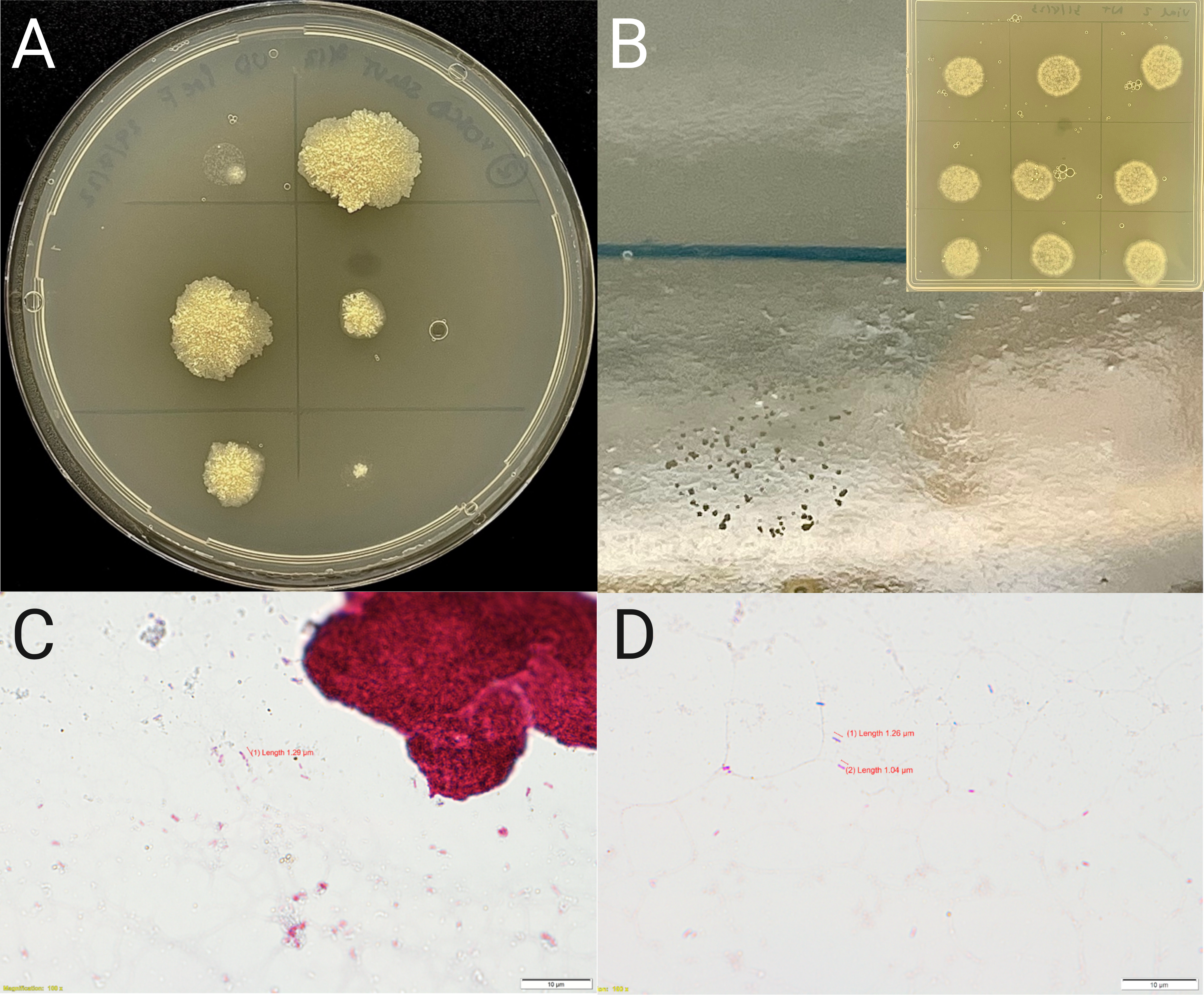

Supplement: S1 Fig — Pre-filtration (A) and post-filtration (B) spot plates of M. ulcerans JKD8049; pre-filtration spots demonstrate irregular colonies, due to the presence of clumps, which quickly overgrow other colonies and make enumeration difficult. Post-filtration spots demonstrate small, separated, countable microcolonies; inset demonstrates homogenous growth of filtered M. ulcerans JKD8049 after 12 weeks (20 μL spots of 9 technical replicates are shown). Panel (C) shows a Ziehl-Neelsen stain of M. ulcerans JKD8049 cultured in orbital shaker with glass beads for 12 weeks before filtration, at 100x magnification; occasional large clumps are visible, with a background of numerous individual bacilli; (D) Ziehl-Neelsen stain of M. ulcerans JKD8049 filtered through a 5 μm pore filter, at 100x magnification. Bacilli are 1.0 to 1.5 μm in length, with no clumps visible in > 30 high power fields. (TIFF) [file pntd.0011979.s001.tiff]
