# Supplementary material for: Mycobacterium ulcerans challenge strain selection for a Buruli ulcer controlled human infection model
Source: PLoS Negl Trop Dis. 2024 May 3;18(5):e0011979. doi: 10.1371/journal.pntd.0011979 (PMC11095734; doi:10.1371/journal.pntd.0011979)
Supplement: S1 Table — (DOCX) [file pntd.0011979.s003.docx]

**S1 Table.** *M. ulcerans* isolates and their respective sequencing reactions.

| \| **Isolate ID** \| **Location** \| **Source** \| **Accession** \| **Bioproject** \| \| --- \| --- \| --- \| --- \| --- \| \| Mm (MMA1) \| Maryland, United States \| Clinical \| CP058277.1 \| PRJNA630296 \| \| Agy99 \| Ghana \| Clinical \| SAMN02603346 \| PRJNA16230 \| \| Mu_JH128_1990 \| Queensland, Australia \| Clinical \| SRR6346308 \| PRJNA421048 \| \| Mu_V75_2004 \| Darwin, Northern Territory, Australia \| Clinical \| SRR6346269 \| PRJNA421048 \| \| Mu_85_2005 \| Gippsland, Victoria, Australia \| Clinical \| SRR6346339 \| PRJNA421048 \| \| Mu_V71_2005 \| Papua New Guinea \| Clinical \| SRR6346268 \| PRJNA421048 \| \| Mu_167_2008 \| Point Lonsdale, Victoria, Australia \| Clinical \| SRR6346333 \| PRJNA421048 \| \| *M._liflandii* 128FXT \| California, United States \| Frog \| SAMN02603618 \| PRJNA20227 \| \| JKD8049 \| Point Lonsdale, Victoria, Australia \| Clinical \| CP085200.1 \| PRJNA771185 \| \| DMG2211762 \| Benin \| Clinical \| SAMN40596023 \| PRJNA771185 \| \| DMG2211768 \| Ghana \| Clinical \| SAMN40596024 \| PRJNA771185 \| \| DMG2211764 \| China \| Clinical \| SAMN40596025 \| PRJNA771185 \| \| DMG2211766 \| Belgium \| Fish \| SAMN40596026 \| PRJNA771185 \| |
| --- | --- | --- | --- | --- | --- | --- | --- | --- | --- | --- | --- | --- | --- | --- | --- | --- | --- | --- | --- | --- | --- | --- | --- | --- | --- | --- | --- | --- | --- | --- | --- | --- | --- | --- | --- | --- | --- | --- | --- | --- | --- | --- | --- | --- | --- | --- | --- | --- | --- | --- | --- | --- | --- | --- | --- | --- | --- | --- | --- | --- | --- | --- | --- | --- | --- | --- | --- | --- | --- | --- |
