# Supplementary material for: Mycobacterium ulcerans challenge strain selection for a Buruli ulcer controlled human infection model
Source: PLoS Negl Trop Dis. 2024 May 3;18(5):e0011979. doi: 10.1371/journal.pntd.0011979 (PMC11095734; doi:10.1371/journal.pntd.0011979)
Supplement: S2 Table — (DOCX) [file pntd.0011979.s004.docx]

**S2 Table**. *M. ulcerans* JKD8049 mean CFU/mL in SMVT over time using orbital shaking cultures with glass beads, incubated at 30°C. 6 biological replicates were tested (Rep 1-6).

|  | **48 hours** | **2 weeks** | **4 weeks** | **6 weeks** | **8 weeks** | **10 weeks** | **12 weeks** |
| --- | --- | --- | --- | --- | --- | --- | --- |
| Rep 1 | 6.30E+04 | 1.03E+06 | 1.33E+06 | 2.80E+06 | 2.27E+06 | 1.40E+06 | 9.33E+05 |
| Rep 2 | 8.33E+04 | 6.67E+05 | 5.93E+06 | 3.83E+06 | 2.27E+06 | 2.07E+06 | 2.33E+05 |
| Rep 3 | 5.00E+04 | 1.43E+06 | 7.33E+06 | 5.33E+06 | 3.57E+06 | 3.30E+06 | 2.73E+06 |
| Rep 4 | - | 6.00E+05 | 2.76E+06 | 2.83E+06 | 2.60E+06 | 1.43E+06 | 7.33E+05 |
| Rep 5 | - | 9.33E+05 | 4.73E+06 | 3.83E+06 | 1.80E+06 | 1.10E+06 | 6.33E+05 |
| Rep 6 | - | 2.03E+06 | 5.60E+06 | 2.00E+06 | 2.13E+06 | 1.57E+06 | 6.67E+05 |
| Mean | 6.54E+04 | 1.12E+06 | 4.61E+06 | 3.75E+06 | 2.44E+06 | 1.81E+06 | 9.88E+05 |
| SD | 1.68E+04 | 6.89E+05 | 2.33E+06 | 2.01E+06 | 8.93E+05 | 9.79E+05 | 8.48E+05 |
| *p* | N/A | *Comparator* | 0.031 | 0.046 | 0.382 | 0.964 | 0.330 |
