# Supplementary material for: Mycobacterium ulcerans challenge strain selection for a Buruli ulcer controlled human infection model
Source: PLoS Negl Trop Dis. 2024 May 3;18(5):e0011979. doi: 10.1371/journal.pntd.0011979 (PMC11095734; doi:10.1371/journal.pntd.0011979)
Supplement: S3 Table — (DOCX) [file pntd.0011979.s005.docx]

**S3 Table.** Mean CFU/mL and 95% confidence interval before and after filtration of orbital shaking cultures with glass beads.

|  | **Incubation duration** | **Pre-filtration CFU/ml (mean)** | **Post-filtration CFU/ml (mean)** | **Filtration loss (%)** |
| --- | --- | --- | --- | --- |
| JKD8094  Benin | 12 weeks | 7.30 x 10^4^ | 3.47 x 10^4^ | 48% |
|  | 95% CI | 6.86 - 7.74 x 10^4^ | 2.65 - 4.28 x 10^4^ |  |
| NM20/02  Ghana | 5 weeks | 4.83 x 10^5^ | 8.97 x 10^4^ | 81% |
|  | 95% CI | 4.30 - 5.37 x 10^5^ | 8.13 - 9.80 x 10^4^ |  |
| JKD8095  China | 5 weeks | 2.70 x 10^5^ | 4.60 x 10^4^ | 85% |
|  | 95% CI | 2.38 - 3.02 x 10^5^ | 3.96 - 5.24 x 10^4^ |  |
| JKD8097  Belgium | 5 weeks | 4.0 x 10^5^ | 2.6 x 10^5^ | 35% |
|  | 95% CI | 3.2 - 4.8 x 10^5^ | 2.1 - 3.1 x 10^5^ |  |
| JKD8049 Australia | 12 weeks | 2.08 x 10^4^ | 5.48 x 10^3^ | 74% |
|  | 95% CI | 1.68 - 2.49 x 10^4^ | 5.17 - 5.80 x 10^3^ |  |
