# Supplementary material for: Mycobacterium ulcerans challenge strain selection for a Buruli ulcer controlled human infection model
Source: PLoS Negl Trop Dis. 2024 May 3;18(5):e0011979. doi: 10.1371/journal.pntd.0011979 (PMC11095734; doi:10.1371/journal.pntd.0011979)
Supplement: S4 Table — TTP: Time-to-positivity from thawing to final CFU enumeration; ‘% loss’ is the percentage reduction in viable cell count compared to prior to cryopreservation, accounting for dilution in glycerol storage media; 95% CI: 95% Confidence Interval. (DOCX) [file pntd.0011979.s006.docx]

**S4 Table.** Mean CFU/mL, 95% confidence interval and viability loss compared to expected CFU/ml prior to glycerol storage in -80°C (accounting for dilution factor).

|  | **JKD 8094** | **NM20/02** | **JKD 8049** | **JKD 8095** | **JKD 8097** |
| --- | --- | --- | --- | --- | --- |
| TTP: | 10 weeks | 10 weeks | 6 weeks | 3 weeks | 2 weeks |
| **0 hr**  95% CI  % loss | **2.17** x **10^3^**  0.95 - 3.38 x10^3^  81% | **2.67**  x **10^4^**  1.81 - 3.52 x10^4^  11% | **1.83**  x **10^3^**  1.68 - 1.98 x10^3^  0% | **3.23** x **10^3^**  2.83 - 3.64 x10^3^  79% | **2.43** x **10^4^**  1.74 - 3.13 x10^4^  72% |
| **1 hr**  95% CI  % loss | **2.20** x **10^3^**  1.27 - 3.13 x10^3^  81% | **2.37**  x **10^4^**  1.74 - 3.00 x10^4^  21% | Not tested | **3.40** x **10^3^**  2.84 - 3.96 x10^3^  78% | **2.53** x **10^4^**  2.14 - 2.92 x 10^4^  71% |
| **2 hrs**  95% CI  % loss | **1.53** x **10^3^**  0.90 - 2.17 x10^3^  87% | **3.00**  x **10^4^**  2.56 - 3.44 x10^4^  0% | **1.76** x **10^3^**  1.66 - 1.85 x10^3^  4% | **3.77** x **10^3^**  3.21 - 4.32 x 10^3^  75% | Uncountable |
| **4 hrs**  95% CI  % loss | Not tested | Not tested | **1.66** x**10^3^**  1.54 - 1.78 x 10^3^  9% | Not tested | Not tested |

TTP: Time-to-positive from thawing to final CFU enumeration; ‘% loss’ is the percentage reduction in viable cell count compared to prior to cryopreservation, accounting for dilution in glycerol storage media; 95% CI: 95% Confidence Interval.
