# Supplementary material for: Mycobacterium ulcerans challenge strain selection for a Buruli ulcer controlled human infection model
Source: PLoS Negl Trop Dis. 2024 May 3;18(5):e0011979. doi: 10.1371/journal.pntd.0011979 (PMC11095734; doi:10.1371/journal.pntd.0011979)
Supplement: S1 Text — (DOCX) [file pntd.0011979.s007.docx]

**Supplementary text**

**S1 Text: Mycolactone mass spectrometry**

Mycolactone mass spectrometry data were collected on a Waters Synapt G2 in DDA mode using a Kinetex 1.7 µm EVO C18 100 Å, 100 x 1.0 mm column (Phenomenex) and run using the following gradient with water + 0.1% formic acid (A) and acetonitrile + 0.1% formic acid (B): 5 min, 10% B, 19 min, 100% B, 22 min 100% B, 22.5 min 10% B, 25 min 10% B. Raw mass spectrometry data files are available at: [https://doi.org/10.26188/c.7161469.v1](https://secure-web.cisco.com/1RE7zyRjsnWlZL5leODajDmvMLomS6p2YqKL9FYah7epo2osi46oKnhOyRhJLVuJozyAR9GQ9O_Ov3fMQdswWzrdjzz-NOPb0lHnexkGi2E3fbWa8Yw9oekyhvYflzIafOawU_UyVlGM87F2ZUTcsRMTRFv0fbr5nggkeDZ02lgj5j6C7X13zAQ_T7vhZYRgaE-q6I-UjaTv0q2K1I3yEUmB7K9eqJ3ICC6bWt8Hyfa1eseqnWTl2XlCdbiiZ8a1M8gJgUdoZh2ZOWeO2NBHfEPJx6ukwWy9nlfqvZi5qtotzBO0bBoJMqrpQvtiQZD3-/https%3A%2F%2Fdoi.org%2F10.26188%2Fc.7161469.v1)
